# Patient experience in systemic lupus erythematosus: development of novel patient-reported symptom and patient-reported impact measures

**DOI:** 10.1186/s41687-018-0028-7

**Published:** 2018-02-22

**Authors:** S. D. Mathias, P. Berry, J. De Vries, K. Pascoe, H. H. Colwell, D. J. Chang, A. D. Askanase

**Affiliations:** 1 Health Outcomes Solutions, PO Box 2343, Winter Park, FL 32790 USA; 2 GlaxoSmithKline, Philadelphia, PA USA; 3 GlaxoSmithKline, London, UK; 4 Columbia University Medical Center, New York, NY USA

**Keywords:** Patient experience, Patient-reported outcomes, Questionnaire, Systemic lupus erythematosus, Symptoms

## Abstract

**Background:**

Comprehensive assessment of systemic lupus erythematosus (SLE) and its treatment requires patient-reported outcome (PRO) measures to capture impacts and fluctuating symptoms. The objective of this study was to develop PROs, in accordance with the Food and Drug Administration (FDA) PRO Guidance, to assess fluctuations in SLE symptoms and its impact.

**Methods:**

Following independent review board approval, six US rheumatology practices recruited patients with SLE to participate in concept elicitation (CE) interviews, in order to identify important SLE symptoms and their impacts. The SLE Symptom Severity Diary (SSD) and SLE Impact Questionnaire (SIQ) were drafted based on CE interview results and clinician input. The PROs were revised based on patient feedback from cognitive debriefing (CD) interviews, clinician feedback, and a translatability assessment.

**Results:**

Forty-one patients completed CE interviews. Commonly-reported symptoms included fatigue (98%), joint pain (93%), and rash (88%). The most frequently reported impact was difficulty with chores/housework (61%). Eighteen patients completed CD interviews. The PROs were considered comprehensive, clear, and relevant.

The SSD contains 17 items assessing energy/vitality, joint and muscle pain/stiffness/swelling, flu-like symptoms, cognition, numbness/tingling, skin symptoms and hair loss using an 11-point numeric response scale and a 24-h recall period (with the exception of hair loss). It also evaluates steroid status and dose. The SIQ contains 50 items, uses a 5-point Likert scale and a 7-day recall period, to assess disease impacts including patients’ ability to make plans, work, and physical/social/emotional functioning.

**Conclusion:**

The SSD and SIQ are comprehensive SLE-specific PROs developed in accordance with the FDA PRO Guidance. Following assessment of their measurement properties, they may be useful in clinical studies and clinical practice to measure fluctuations in, and the impact of, symptoms in patients with SLE.

## Background

Systemic lupus erythematosus (SLE) is a complex, chronic disease characterized by fluctuating symptoms, flares and remissions [1]. As recognized by the Food and Drug Administration (FDA) [2] and European Medicines Agency (EMA) [3], clinical and laboratory measures of SLE disease severity do not assess the impact of symptom- and treatment-related effects of SLE on patients’ daily lives. Therefore, reliable patient-reported outcome (PRO) measures are important tools in both SLE clinical studies and clinical practice to enable comprehensive assessment of the disease and its treatments.

The FDA PRO Guidance recommends using an iterative process, including concept elicitation (CE) and cognitive debriefing (CD) interviews, in order to gain substantial input from patients who are representative of the target population in which the tool is intended to be used [4]. To determine the content of the PRO, CE interviews assist in identifying the concepts that are most important and relevant to the target population [5]. Through CD interviews, drafts of the PRO should be evaluated by patients to assess their understanding of the PRO and to gain feedback on the content, format, recall period, and response options [6]. In combination with results from CD interviews, clinician assessment of the PRO should be used to revise and refine the PRO.

When using a PRO endpoint in a clinical trial as an assessment of treatment benefit, the FDA requires extensive documented evidence that clearly demonstrates the PRO development process followed best measurement science, that the tool is content valid in the target population, and that it has adequate measurement properties, a scoring algorithm and a responder definition (i.e., the individual patient PRO score change over a predetermined time period that should be interpreted as a treatment benefit) [4–6]. The required evidence can be summarized in a PRO Evidence Dossier, the elements of which have been described previously [4–7]. For our intended clinical trial, a PRO measure of SLE symptoms and impacts is required, which includes concepts that are evaluative in nature, and could potentially be impacted by treatment during the course of an interventional clinical trial. Therefore, this study aimed to develop comprehensive PRO measures, primarily for use in clinical research and, following further development, for use in clinical practice to assess symptoms and impacts of SLE.

In the first part of this study, a detailed literature review identified numerous generic and disease-specific PROs used in patients with SLE; however, none were found to meet our needs, either due to a lack of evidence to satisfy the FDA requirements (many were developed prior to the FDA PRO Guidance) or due to inadequate assessment of all symptoms and impacts of SLE that are likely to be impacted by treatment. Therefore, the second part of this study aimed to develop comprehensive PRO measures to assess symptoms and impacts of SLE.

## Methods

### Study design

**Fig. 1:**
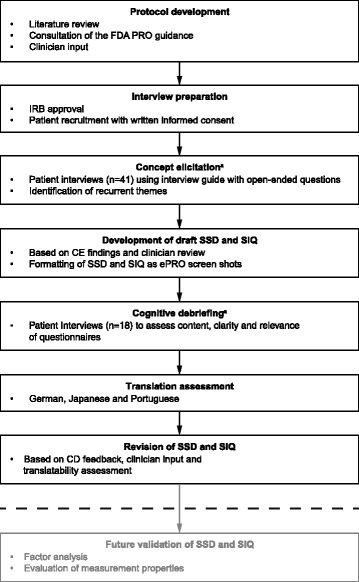
PRO development process. ^a^Distinct groups of patients participated in the CE and CD interviews. CD, cognitive debriefing; CE, concept elicitation; ePRO, electronic patient reported outcome; FDA, Food and Drug Administration; IRB, internal review board; PRO, patient-reported outcome; SIQ, SLE Impact Questionnaire; SSD, SLE Symptom Severity Diary

A detailed research protocol was developed in accordance with best measurement science (Fig. 1) [4–6]. Independent review board (IRB) approval (Copernicus Group) was obtained.

### Literature review

A comprehensive literature review was undertaken in 2012 (and updated in 2013) to determine if there was an available PRO developed with significant input from patients with SLE that would capture the daily variability in symptoms and disease impacts (data on file). The literature search identified published articles (PubMed, EMBASE), conference abstracts (scientific meeting abstracts from the American College of Rheumatology [ACR] and International Society for Quality of Life Research annual scientific meetings from the previous 2 years) and clinical studies from the previous 5 years (ClinicalTrials.gov database) that included a PRO or disease activity index and patients with SLE. Since the initial literature search was undertaken, PubMed had been monitored (2013–present) for any new, relevant articles.

### Study population

Between May and July 2014, a convenience sample of patients was recruited from six rheumatology practices from a wide geographic area of the US (California, New Jersey, Michigan, Georgia, Florida, and Virginia); recruitment took place at the clinic or via telephone. Patients were US residents, 18–75 years of age, had a clinical diagnosis of SLE according to the ACR classification criteria [8, 9], were able to speak and read English, and provided written informed consent. Patients with a medical or psychiatric condition or those receiving treatment for a condition that causes cognitive or other impairments, which the investigator judged would interfere with study participation, were excluded. Patients were screened and selected to ensure a diverse population was recruited in terms of demographic and clinical characteristics, including African Americans, males, individuals in paid employment, and with a wide range of age, Safety of Estrogens in Lupus Erythematosus National Assessment-Systemic Lupus Erythematosus Disease Activity Index (SELENA-SLEDAI) scores, and organ involvement. Patients took part in either the CE or CD interviews, but not both.

Physicians completed a clinical case report form for each patient enrolled to provide their clinical status and details of the patient’s current and previous (past 2 years) SLE treatments. Physicians determined the patient’s SLE disease severity based in part on SELENA-SLEDAI scores, Systemic Lupus International Collaborating Clinics/ACR (SLICC/ACR) damage index, or both (when available), and rated it (mild, moderate, or severe) over the previous 6 months.

All practices received an honorarium, and upon completion of the interview each patient received a gift card for their participation.

### Patient interviews and PRO development

CE and CD interviews were conducted separately, using two separate samples of patients with SLE.

#### CE interviews

CE interviews elicited important concepts from individuals with SLE, specifically about SLE-related symptoms and impacts. Face-to-face interviews were conducted by trained interviewers using a semi-structured interview guide developed for this study. To ensure all relevant topics were discussed, the interview guide included broad, open-ended questions such as, *‘What symptoms, if any, do you ever experience as a result of your lupus?’* and *‘How does having lupus affect your day-to-day life?’* Patients were questioned on the overall impact of SLE, symptoms of SLE, day-to-day impacts of SLE, and their experience taking steroids (if recently or currently receiving steroids). Development of the accompanying steroid-specific PRO measure is reported elsewhere [10]. Interviews were conducted until concept saturation (the point at which no new concepts emerged from the interviews) was reached. Based on analysis of the CE interview transcripts and clinician input, the SLE Symptom Severity Diary (SSD) and the SLE Impact Questionnaire (SIQ) were drafted.

#### CD interviews

CD interviews, using both debriefing and a “think aloud” approach, assessed patient understanding of the draft PROs and evaluated the content, clarity and relevance of the questions, response options and recall periods. A second sample of eligible patients with SLE was recruited from four of the six practices. Just prior to the face-to-face interviews, patients completed paper versions of the PROs, formatted to look like electronic screen shots, as future administration of the tools using an electronic device (ePRO) is planned. CD interviews were conducted using a semi-structured interview guide developed for the study, with questions such as, ‘*Looking at question 1 (feeling tired or lacking energy), how do you define feeling tired? What about lacking energy? What is the difference between these terms? Why would you say that? Do you think we need to include both terms or would you delete one? (If delete one), Which would you omit? Why? Do you have any other suggestions about how this question could be revised? If yes, please describe. Why do you think that is clearer?*’ All patients (with the exception of one) were asked about both PROs, but they were not asked to evaluate every item; the items perceived to be the most challenging were tested and to avoid the interviews becoming tedious for the patients, they were not asked to evaluate questions that were very similar to each other. Multiple rounds of CD interviews were conducted. After each round of interviews, the responses were reviewed and the PROs were modified accordingly. The revised drafts were then used in the next round of interviews.

To enable worldwide use of the PROs, translators assessed whether the questionnaires could be readily translated into three etymologically distinct languages (German, Japanese, and Portuguese). Based on the CD interviews, clinician input, and the translatability assessment, both PROs were revised.

### Data analyses

All interviews were recorded and transcribed for analysis; patient identifiable information was not included in any transcripts or analyses to ensure patient confidentiality. All data were held in strict confidence in accordance with local, state, and federal law.

Interview data were coded using MAXQDA (Verbi GmbH, Berlin, Germany). A coding dictionary was developed and used in the thematic analysis. Each transcript was coded by one coder, then reviewed, summarized, and analyzed by a second coder. Similar concepts/themes were grouped together to allow for quantitative analysis. In addition to analyzing the data from the code book, representative quotes were reviewed. To the extent possible, language provided by patients was utilized to develop the items in both the SSD and SIQ. In this way, the rich patient narratives were converted into the final list of symptoms included in the SSD and the impacts described in the SIQ.

Safety was not assessed in the study; however, if patients reported an adverse event during the interview, they were instructed to report it to their healthcare provider; if relevant this was also reported to the study sponsor.

## Results

### Literature review

The literature review identified 68 PROs that had previously been used for evaluating patients with SLE; Table 1 summarizes the characteristics of PROs that matched some of the desired criteria for this study. The most commonly-cited PRO was the Medical Outcomes Short Form-36 (SF-36), which is a generic measure of health-related quality of life (HRQoL), provides a broad measure of functioning and well-being, but was not developed in an SLE population and does not assess all concepts that are important to patients with SLE [11]. Symptom-specific measures such as the Functional Assessment of Chronic Illness Therapy-Fatigue (FACIT-Fatigue) [12], the Beck Depression Inventory [13], and the Brief Pain Inventory [14] were used to measure commonly-reported symptoms of SLE [15]. However, these measures were not developed in an SLE population and do not include all relevant SLE symptoms. Existing SLE-specific PRO measures such as the Lupus Quality of Life questionnaire (LupusQoL) [16] and the Lupus Patient Reported Outcome questionnaire (LupusPRO) [17] can be used to measure HRQoL, but may not be suitable for frequent self-administration and do not include a comprehensive assessment of symptoms. The Lupus Impact Tracker (LIT) [18] was developed for use in clinical practice, from the LupusPRO, as a brief measure to assess the impact of SLE. The Systemic Lupus Activity Questionnaire (SLAQ) [19], which was developed as a self-report version of the Systemic Lupus Activity Measure (SLAM), is used to screen for possible flares requiring further evaluation. Most SLE-specific PROs utilize a relatively long recall period of at least 4 weeks, so are unlikely to capture daily variability in symptoms and may have a greater risk of recall bias [16–18]. The SLE-specific Quality of Life (SLEQOL) instrument was developed by health professionals and only subsequently was the content assessed by patients [20].

**Table 1:** Summary of commonly used SLE PROs and characteristics of the SSD and SIQ

PRO	Concept measured	Intended use	Content	Recall period
SF-36^a^ [11]	HRQoL	General population surveys and clinical trials to estimate disease burden and compare disease-specific benchmarks with general population norms	Generic measure of physical and mental functioning with 36 items contributing to 8 subscales: physical functioning, role-physical, bodily pain, general health, vitality, social functioning, role-emotional, and mental health	Standard version: 4 weeks; acute version: 1 week
LupusQoL [16]	HRQoL	Adults with SLE in clinical trials and clinical practice	34 items in 8 domains: physical health, pain, planning, body image, burden to others, intimate relationships, emotional health, and fatigue	4 weeks
LupusPRO [17]	HRQoL	US patients with SLE in clinical trials and clinical practice	30 items in 8 HRQoL domains: lupus symptoms, lupus medications, cognition, procreation, physical health, emotional health, pain-vitality and body image. 13 items in 3 non-HRQoL domains: desires goals, available social support and coping and satisfaction with medical care	4 weeks
L-QoL [29, 32]	HRQoL, impacts	Patients with SLE in clinical trials and clinical practice	25 items investigating fatigue and impacts including daily activities, emotional/psychological, social functioning, and relationships	At the moment
SLEQOL [33]	HRQoL	Patients with SLE in clinical trials and clinical practice	40 items in 6 subsections: physical functioning, activities, symptoms, treatment, mood and self-image	1 week
SSC [21]	Symptoms	Patients with SLE in clinical trials and clinical practice	38 items measuring disease-related and treatment-related symptoms and their burden	1 month
SLAQ^b^ [19]	Symptoms	Tracking and screening of patients with SLE in epidemiologic studies	24 items measuring symptoms of disease activity	3 months
Lup-QOL [32, 33]	HRQoL	Patients with SLE in clinical trials and clinical practice	Incorporates the Medical Outcomes Study SF-36 and FACIT-Fatigue. Includes 19 items with generic and disease-specific components including symptoms and interference, cognitive and confidence and planning	Varies according to the item
SSD	Symptoms	Patients with SLE in clinical trials and practice	17 items measuring SLE symptoms (including: energy/vitality, joint or muscle pain/stiffness/swelling, cognition and skin symptoms), and current steroid dose	1 day
SIQ	Impact	Patients with SLE in clinical trials and practice	50 items measuring SLE impacts including daily activities; social, physical and emotional functioning; and work productivity	7 days

^a^With the exception of the SF-36 and Lup-QOL, all other measures utilized input from patients with SLE in their development; ^b^SLAQ is the only screener developed for use in the US with English-speaking patients

*L-QoL* Systemic Lupus Erythematosus Quality of Life Questionnaire, *Lup-QOL* Lupus-Quality of Life, *PRO* patient-reported outcome, *SF-36* Short Form-36, *SIQ* SLE Impact Questionnaire, *SLAQ* Systemic Lupus Activity Questionnaire, *SLE* systemic lupus erythematosus, *SLEQOL* Systemic Lupus Erythematosus Quality of life Instrument, *SSC* SLE Symptom Checklist, *SSD* Symptom Severity Diary

Many of the PROs were developed prior to the release of the FDA PRO Guidance (2009) [4] and therefore the required documented evidence of their development is not available. Existing measures were also found to be insufficient as their development lacked substantial, documented input from patients with SLE (e.g., SF-36, FACIT-Fatigue), their content focused on HRQoL or concepts not expected to be directly impacted by treatment (such as body image) instead of symptoms and impacts (e.g., LupusQoL, LupusPRO), and/or their structure and recall period were not suitable for capturing the frequently fluctuating nature of SLE. Recently, the use of items in the Patient Reported Outcomes Measurement Information System (PROMIS^®^) item bank [21, 22] has been explored in studies of SLE [23, 24]. Although the PROMIS item bank contains items that are relevant to SLE (e.g., fatigue and pain), it is not comprehensive, and further work is needed to evaluate the specific items and associated measurement properties for use in patients with SLE. It was, therefore, concluded that the development of novel measures to evaluate symptoms and impacts would be of value for both clinical research and clinical practice.

### CE interviews

CE interviews were completed by 41 patients (3–10 patients from each study site): 95% female, 55% Caucasian, 25% African American (Table 2). Mean (standard deviation [SD]) time since SLE diagnosis was 8.56 (8.13) years; according to physician assessment, 54%, 44%, and 2% of patients had mild, moderate, and severe disease, respectively.

**Table 2:** Demographic and clinical characteristics of patients

	CE interviews (*n* = 41)	CD interviews (*n* = 18)
Gender, n (%)		
Male	2 (5)	1 (6)
Female	39 (95)	17 (94)
Mean age, years (SD)	47.7 (12.6)	50.8 (14.4)
Ethnicity, n (%)		
Caucasian	22 (55)	7 (39)
African American	10 (25)	11 (61)
Latino/Hispanic	5 (12)	0
Other	2 (5)	0
Asian	1 (2)	0
Marital status, n (%)		
Married	16 (39)	5 (28)
Lives with partner	0	1 (6)
Widowed/divorced/separated	8 (19)	6 (33)
Single/never married	17 (42)	5 (28)
No response	0	1 (6)
Employment, n (%)		
Not currently working for pay	11 (27)	2 (11)
Working full time	11 (27)	4 (22)
Working part time	1 (2)	2 (11)^a^
Retired	6 (15)	5 (28)
Disabled	0	5 (28)
Other	12 (29)	0
Mean time since SLE diagnosis, years (SD)^b^	8.56 (8.13), range < 1–35 (n = 38)	11.28 (10.38), range 1–38 (n = 14)
Mean SELENA-SLEDAI^c^ (SD)	6.02 (3.60) (n = 37)	6.06 (2.53)
Mean SLICC/ACR damage index^c^ (SD)	5.96 (1.74) (n = 25)	6.00 (1.83) (n = 10)
SLE severity, n (%)		
Mild	22 (54)	9 (50)
Moderate	18 (44)	9 (50)
Severe	1 (2)	0
Current SLE treatment^d^, n (%)		
Hydroxychloroquine	29 (71)	14 (78)
Corticosteroids	19 (46)	10 (56)
NSAIDs	7 (17)	2 (11)
Belimumab	6 (15)	2 (11)
Methotrexate	4 (10)	3 (17)
Concomitant condition^e^, n (%)		
Fibromyalgia	10 (24)	4 (22)
Osteopenia/osteoporosis	9 (22)	5 (28)
Anxiety	9 (22)	3 (17)
Hypertension	8 (20)	4 (22)
Depression	7 (17)	2 (11)
Rheumatoid arthritis	5 (12)	1 (6)
Asthma	4 (10)	2 (11)
Osteoarthritis	4 (10)	4 (22)
Sjögren’s syndrome	4 (10)	2 (11)
Lupus nephritis	3 (7)	4 (22)
Hyperlipidemia/hypercholesterolemia	2 (5)	3 (17)
Vasculitis	2 (5)	2 (11)
Other renal diseases (non-lupus)/ESRD	1 (2)	2 (11)
Congestive heart failure	0	2 (11)

^a^One patient was also a student; ^b^if patients had moved practice since initial SLE diagnosis it was not always possible to precisely determine their date of diagnosis; ^c^when data were available; ^d^some patients were receiving more than 1 treatment; ^e^reported by ≥10% of patients

*CE* concept elicitation, *CD* cognitive debriefing, *ESRD* end stage renal disease, *NSAIDs* non-steroidal anti-inflammatories, *SD* standard deviation, *SELENA-SLEDAI* Safety of Estrogens in Lupus Erythematosus National Assessment-Systemic Lupus Erythematosus Disease Activity Index, *SLE* systemic lupus erythematosus, *SLICC/ACR* Systemic Lupus International Collaborating Clinics/American College of Rheumatology

The most frequently-reported symptoms were fatigue (98%, *n* = 40/41), joint pain (93%, *n* = 38/41), rash (88%, *n* = 36/41), swelling of hands, fingers, feet or legs (80%, *n* = 33/41), and joint stiffness/cramps (80%, n = 33/41) (Table 3). One patient described fatigue as, *“It’s a feeling inside, where sometimes you’re so tired you just don’t want to even breathe. It’s just an effort to move your arms.”* Concept saturation, the point at which no new concepts are mentioned by subsequent patients, was reached by the 26th of 41 interviews.

**Table 3:** Frequency of symptoms reported during CE interviews (n = 41) when patients were asked to describe what symptoms they experience as a result of lupus

Symptom	Reported spontaneously, n (%)	Reported in response to specific probing, n (%)	Total, n (%)^a^
Fatigue	35 (85)	5 (12)	40 (98)
Joint pain	34 (83)	4 (10)	38 (93)
Rash	19 (46)	17 (41)	36 (88)
Swelling of hand, fingers, feet or legs	16 (39)	17 (41)	33 (80)
Joint stiffness/cramps	21 (51)	12 (29)	33 (80)
Cognitive/focus/memory issues	9 (22)	17 (41)	26 (63)
Headaches	9 (22)	14 (34)	23 (56)
Muscle pain/achiness	15 (37)	0	15 (37)
Fevers/hot flashes	14 (34)	0	14 (34)
Dry/itchy skin	9 (22)	0	9 (22)
Chills	9 (22)	0	9 (22)
Decreased appetite/weight loss	8 (20)	0	8 (20)
Hair loss	8 (20)	0	8 (20)
Raynaud’s	8 (20)	0	8 (20)
Mouth sores	7 (17)	0	7 (17)
Numbness/tingling	7 (17)	0	7 (17)
Weakness/decreased muscle strength	3 (7)	4 (10)	7 (17)
Dry eyes/vision issues	6 (15)	0	6 (15)
Nausea/vomiting	6 (15)	0	6 (15)
Shortness of breath	6 (15)	0	6 (15)
Coughing/wheezing	4 (10)	0	4 (10)
High blood pressure	3 (7)	1 (2)	4 (10)
Muscle stiffness/cramps	4 (10)	0	4 (10)
Rapid heart rate	4 (10)	0	4 (10)
Weight gain	0	4 (10)	4 (10)
Dizziness	3 (7)	0	3 (7)
Bladder issues	2 (5)	0	2 (5)
Insomnia	2 (5)	0	2 (5)
Night sweats	2 (5)	0	2 (5)
Nose bleeds	2 (5)	0	2 (5)
Tremors/twitching	2 (5)	0	2 (5)
Bone pain	1 (2)	0	1 (2)
Dry mouth	1 (2)	0	1 (2)
Panic attacks	1 (2)	0	1 (2)
Seizures	1 (2)	0	1 (2)

^a^Total % may be higher due to rounding

*CE* concept elicitation

When asked how SLE impacts their day-to-day life, patients reported difficulty with chores/housework (61%, *n* = 22/36), leisure activities (39%, *n* = 14/36), driving (39%, *n* = 14/36), sleeping (39%, *n* = 14/36), cooking (31%, *n* = 11/36), taking care of children (22%, *n* = 8/36), and washing, brushing, or drying their hair (22%, n = 8/36). SLE was also found to impact patients’ ability to work. Of the patients not currently working outside the home (62%, *n* = 24/39), 91% (*n* = 20/22 asked) reported that they did not work because of SLE. Of those who currently worked for pay, 33% (*n* = 5/15) reported missing work due to SLE, and 80% (*n* = 12/15) stated that SLE had an impact on work productivity.

**Fig. 2:**
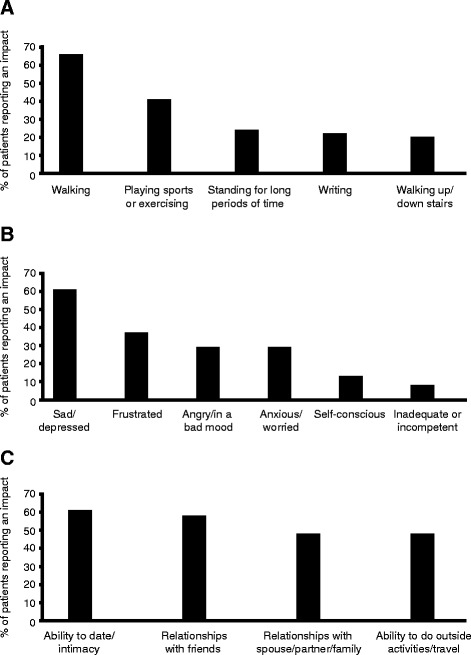
Patient-reported impact of SLE on functioning according to the CE interviews. A. Physical functioning (*n*=41); B. Emotional functioning (*n*=38); C. Social functioning (*n*=31).

The most commonly-reported impacts on physical, emotional, and social functioning were decreased ability to walk (66%, *n* = 27/41), feeling sad/depressed (61%, *n* = 23/38), and decreased ability to date or be intimate with a partner (61%, *n* = 19/31) (Fig. 2). Representative patient quotes include, “*I can’t do as much as I used to do. I can’t walk as far as I used to. You know I have to park like in the disabled parking”*; *“I think the depression is definitely due to lupus. I don’t think in general I am depressed. I’m positive about most things. I enjoy company of other people. What depresses me is when I just can’t move and then I’m more isolated”; “I was very social. I ran organizations. I had a lot of friends. I will say I have three friends who contact me consistently now, that’s it”* and “*Well, that’s why I’m getting the divorce. Because of the impact, I don’t feel sexual*”.

When asked what the single worst thing about having SLE is, the most common answers were the inability to do what they wanted to do (44%, *n* = 17/39), that there is no cure (13%, *n* = 5/39), fatigue 10% (*n* = 4/39), pain (10%, n = 4/39), rash (8%, *n* = 3/39), and the unpredictable nature of lupus (8%, n = 3/39).

### Drafting of the PROs, CD interviews, and questionnaire revisions

Based on analyses of the CE interview transcripts, draft versions of the SSD and SIQ were developed and reviewed by two rheumatologists.

CD interviews to assess the content and clarity of the draft questionnaires were completed by a second group of 18 patients with SLE: 94% female, 39% Caucasian, and 61% African American (Table 2). The mean (SD) time since SLE diagnosis was 11.28 (10.38) years; 50% of patients had mild SLE and 50% had moderate SLE. At the final round of CD interviews there was little new information gained.

#### SLE symptom severity diary

The majority of instructions and questions were found to be clear and concise and could be paraphrased correctly by most patients. All patients asked (*n* = 14/14) reported it was easy to complete the questionnaire, and that the ordering of the items was appropriate. On average, it took 6 min (range 2–15 min) to complete the questionnaire.

Eighty-two percent (*n* = 14/17) of patients asked thought all items were relevant to patients with SLE. Forty-three percent (*n* = 3/7) of patients asked did not think there were any missing symptoms. Additional symptoms identified as missing were muscle cramps (*n* = 2), shortness of breath, heart palpitations, dry mouth, difficulty hearing, not feeling attractive, and emotional/physical stress (all n = 1); due to the low rate of endorsement these were not included in the questionnaires.

The draft SSD used an 11-point (0–10) numeric rating scale (NRS; with 0 being “Absent/Did not have” and 10 being “Worst imaginable”). All patients asked (15/15) were able to correctly paraphrase “absent/did not have,” and “Worst imaginable.” All patients asked (10/10) were also able to find an appropriate response to the questions, and 93% (13/14) thought the number of response options was appropriate; one patient suggested using a scale of 0–5 as they thought 0–10 was too broad. As the majority of patients thought the 0–10 response scale was appropriate, and no patients indicated they would prefer a different set of response options, the 11-point NRS was retained.

One of the few substantial changes made to the SSD was that two questions relating to muscle pain/achiness and muscle stiffness were combined and replaced with, “Rate the severity of muscle pain or stiffness at its worst in the past 24 hours?” Other minor changes included making selected words bold to improve the clarity.

As a result of the translatability assessment, minor revisions were made to some questions and the instructions. For example, “achiness” does not translate in German or Portuguese so it was removed (e.g., “Rate the severity of joint pain or achiness at its worst in the past 24 hours?” was revised to “Rate the severity of joint pain at its worst in the past 24 hours?”). Also, the use of “individuals” was replaced with “people” to enable translation into German and Japanese.

The revised SSD contains 17 items that investigate energy/vitality, joint or muscle pain/stiffness/swelling, flu-like symptoms, cognition, numbness/tingling, skin symptoms, hair loss, and steroid status/dose (descriptive only). Sample items are provided in Table 4. In the CE interviews, patients reported daily fluctuations in symptoms. Therefore, a recall period of 24 h was considered to be optimal and was used in the draft measure. During the CD interviews, 77% of patients asked (*n* = 10/13) felt the 24-h recall period was appropriate for all symptoms except hair loss, where a recall period of 4 weeks was used (Table 1). Selection of the 24-h recall period was supported by the FDA PRO Guidance, which states “*PRO instruments that call for patients to rely on memory, especially if they must recall over a long period of time, compare their current state with an earlier period, or average their response over a period of time, are likely to undermine content validity. Response is likely to be influenced by the patient’s state at the time of recall. For these reasons, items with short recall periods or items that ask patients to describe their current or recent state are usually preferable*.” [4] The SSD utilizes an 11-point NRS, which is preferred over a 5- or 7-point Likert scale, as the measure is intended to be administered daily, and as there is some evidence that suggests the NRS is more sensitive than a verbal rating scale in the assessment of pain intensity and hence may be more likely to capture small daily changes in the severity of SLE symptoms [25].

**Table 4:** Sample questions from the SSD and SIQ

Sample questions from the SSD: People with lupus may experience a variety of symptoms. Please indicate how severe the following lupus symptoms were **at their worst** in the **past 24 hours** by selecting **one** number.											
Rate the severity of **feeling tired OR lacking energy** **at its worst** in the past 24 h?	0 Absent/Did not have	1	2	3	4	5	6	7	8	9	10 Worst imaginable
Rate the severity of **joint pain** **at its worst** in the past 24 h?	0 Absent/Did not have	1	2	3	4	5	6	7	8	9	10 Worst imaginable
Rate the severity of **swelling of joints including fingers, hands, elbows, feet or ankles** **at its worst** in the past 24 h?	0 Absent/Did not have	1	2	3	4	5	6	7	8	9	10 Worst imaginable
Rate the severity of **rash** **at its worst** in the past 24 h?	0 Absent/Did not have	1	2	3	4	5	6	7	8	9	10 Worst imaginable
**Sample questions from the SIQ:** **In the past 7 days,** how much did **lupus** impact your ability to **make plans in advance**? (select one response)											
Not at all	A little bit			Somewhat			Quite a bit		Very much		
**In the past 7 days,** how much did **lupus** impact your ability to **participate in leisure activities or hobbies that require you to leave your house** (such as going to a park, going shopping, going to a restaurant, or going to the movies)? (select one response)											
Not at all	A little bit			Somewhat			Quite a bit		Very much		
**In the past 7 days**, how much did lupus impact your ability to **get together with friends**? (select one response)											
Not at all	A little bit			Somewhat			Quite a bit		Very much		
**In the past 7 days,** how much did **lupus** impact your ability to **walk a short distance (approximately one block)**? (select one response)											
Not at all	A little bit			Somewhat			Quite a bit		Very much		
**In the past 7 days,** how much did **lupus** impact your ability to **feel rested enough to get out of bed in the morning**? (select one response)											
Not at all	A little bit			Somewhat			Quite a bit		Very much		

*SIQ* SLE Impact Questionnaire, *SSD* SLE Symptom Severity DiaryThe actual questionnaire uses some bolding and underlining

#### SLE impact questionnaire

Seventeen patients completed CD interviews of the SIQ; due to the length of the questionnaire, it was not feasible to cognitively debrief all items, therefore, items with words or terms that were more complex or could potentially have more than one interpretation were focused upon. For example, patients were debriefed on whether they considered the terms joint pain and joint stiffness to be the same, whether questions referring to both symptoms should be included, whether they should be kept as separate items and if they answered questions referring to them differently.

All patients asked (*n* = 9) were able to correctly paraphrase the instructions, and 89% of patients (*n* = 8/9) had no suggestions to improve their clarity. The majority of patients asked (78%, *n* = 7/9) did not think any questions were unclear. On average, it took 12 min (*n* = 10; range 5–40 min) to complete the questionnaire; 87% of patients asked (*n* = 13/15) felt the questionnaire was easy to complete, and all patients (n = 9/9) felt the question order was appropriate.

Based on clinician input, a recall period of 7 days was chosen for the first draft of the SIQ. During debriefing, 75% (*n* = 6/8) of patients asked reported that they thought about the past 7 days when answering the questions, and 25% (*n* = 2/8) thought about the entire time since they had been diagnosed with SLE. Thus, it was decided that a recall period of the past 7 days would be most appropriate for the SIQ.

Most revisions to the SIQ involved combining similar items or making minor wording changes to enhance translatability. For example, “In the past 7 days, how much did lupus impact your ability to make plans 2–3 days in advance?” and “In the past 7 days, how much did lupus impact your ability to make plans for the future (more than 2–3 days in advance)?” were combined to read, ‘In the past 7 days, how much did lupus impact your ability to make plans in advance?’

The refined SIQ includes 50 items assessing ability to make plans; take care of yourself/others; leisure activities; social functioning; physical functioning; sleep; memory/cognitive issues; work; and emotional functioning (Table 1), using a 5-point Likert-type response scale. The questionnaire assesses the degree of impact SLE has on various aspects of an individual’s life. As such, a numeric rating of this impact would not necessarily be familiar or meaningful to patients. The response options utilized are standard response options that are used in many PRO questionnaires including the FACIT measurement system [12]. In addition, unlike the SSD, which is administered daily, the SIQ is administered weekly, as symptom impacts are less likely to change on a daily basis. Sixty-nine percent of patients asked (*n* = 9/13) thought the time frame of the past 7 days was appropriate. A Likert-type scale is appropriate for detecting these changes in impacts, as it is easy for respondents to understand and still sensitive enough to detect subtle changes [26]. For the majority of items, the response scale ranges from “Not at all” to “Very much”; three items have responses ranging from “None of the time” to “All of the time”. Sample items are provided in Table 4.

## Discussion

Although the literature review identified numerous PROs used in patients with SLE, it identified a need for PROs developed in alignment with the FDA PRO Guidance to assess the fluctuations in symptoms and impacts experienced by patients with SLE. Existing PROs were either designed for different purposes (e.g., assessing HRQoL on a monthly basis, [16, 17] or to screen for possible flares requiring further evaluation [19]), were not developed specifically for patients with SLE [11–14], or did not provide sufficient evidence of development in line with best measurement science. Although the LupusPRO and LIT may have been developed in alignment with best measurement science, with content validity in the target population of interest and adequate measurement properties as required by the FDA, to the best of our knowledge, at the time of this study there was not comprehensive, publicly available, documented evidence that demonstrated this. Furthermore, they were not developed with the intention to support FDA label claims [17, 18]; however, subsequent to the development of the SSD and SIQ the developers of the LupusPRO and LIT have begun the process of submitting an application to the FDA to qualify these measures.

Two PROs, the SSD and SIQ, were developed based on information from literature and patient and clinician input. Patient input obtained during CE interviews regarding the most common symptoms was aligned with current literature; fatigue and joint pain were most common, reported by over 90% of patients [27–29]. The CE interviews also revealed how SLE impacts patients’ physical, emotional, and social functioning; the themes identified formed the basis of the SIQ.

Drafts of the PROs were evaluated by patients through CD interviews. Patients reported that the PROs were clear, comprehensive, and relevant. Patients were able to complete the questionnaires in 6–12 min, which supports their routine use. Minor modifications to the draft PROs were made based on patient feedback, further clinician input, and an assessment of translatability.

Best scientific practices, as described in the FDA PRO Guidance [4], were followed throughout the development of both PROs, including an iterative development methodology and substantial patient input. Sample sizes were sufficient to support their development. Concept saturation was achieved by the 26th CE interview; this demonstrates that no important concepts are missing since no new concepts were raised during the final 15 CE interviews. We are confident that the CD sample size was sufficient and all items were evaluated satisfactorily; by the final round of reviewing there was little new information gained from the interviews.

Both Caucasian and African American patients were interviewed, and patients with a wide range of disease duration were included. However, only one patient with severe SLE completed the CE interview, and no patients with severe SLE completed CD interviews. There were some instances where patients with mild SLE did not believe an item was relevant because they had not experienced it; therefore, it is important that future evaluation of the PROs includes patients with severe SLE. There were only a few male participants enrolled (5% of the CE and 6% of the CD patients), though this is not unexpected, as SLE is approximately nine times more common in women than men [30]. Further, there was no representation of Hispanic or Asian patients with SLE in the CD sample. It may have been possible to obtain a more representative sample if a larger number of patients had been recruited instead of purposely selecting patients to generate a diverse sample for characteristics including age, race, disease severity and time since diagnosis. Background demographic and clinical information was not collected from patients not eligible to participate or who declined to participate in the study, so it is unclear whether our patient population is representative of the total population approached. Another potential limitation of this study is that not all questions were asked of all patients during the cognitive debriefing of the instruments, therefore in some cases the sample size was smaller.

Despite some limitations, primarily related to the characteristics of the study participants, using patient input to inform the content of the PROs enabled the inclusion of concepts that are most important and relevant to patients.

Compared with existing PROs used for patients with SLE, the SSD and SIQ have a number of novel features that will enhance their utility following confirmation of their measurement properties. Importantly they were developed in accordance with the FDA PRO Guidance [4] and may be appropriate in a specified context to support claims in approved medical product labelling. With the exception of the LupusPRO and LIT, many of the existing PROs used by patients with SLE were developed prior to the publication of the FDA PRO Guidance. Compared with generic PROs such as the SF-36, the SSD and SIQ have been specifically developed for patients with SLE; therefore, they are more comprehensive as they capture the full range of impacts and symptoms that are relevant to patients with SLE. Furthermore, as recommended by the FDA PRO Guidance [4] and endorsed by patients in this study, the short recall periods (24 h for the SSD and 7 days for the SIQ) should enable accurate reporting of fluctuations in SLE symptoms and impacts of the disease. Also, both PROs have been developed for electronic administration to enable patients to complete them regularly and with ease, and ensure more accurate reporting while minimizing missing data. Previously, ePROs have been demonstrated to be a feasible and convenient tool for patients with chronic inflammatory disease [31]. Prior to use, it will be necessary to perform exploratory factor analysis for both PROs, in order to confirm the structure of each scale and develop scoring algorithms. Without this information, only item level data can be presented descriptively. In addition, the measurement properties of the questionnaires, including internal consistency reliability, test-retest reliability, construct validity, known groups validity, responsiveness to change over time, and an estimation of what change is clinically meaningful should be evaluated. Development of a responder definition will support the interpretation of scale scores. We assessed the translatability of the PROs into three distinct languages; if it becomes necessary to translate the PROs in to other languages, further modifications may be required to ensure conceptual equivalence.

## Conclusion

In summary, two new PROs, the SSD and the SIQ, have been developed using a robust methodology. Following psychometric testing and adequate demonstration of the required measurement properties, these tools can be used in clinical research and clinical practice to assess the symptoms and impacts experienced by patients with SLE.

## Abbreviations

ACR: American College of Rheumatology
CD: cognitive debriefing
CE: concept elicitation
EMA: European Medicines Agency
ePRO: electronic patient-reported outcome
FACIT-Fatigue: Functional Assessment of Chronic Illness Therapy-Fatigue
FDA: Food and Drug Administration
HRQoL: health-related quality of life
IRB: internal review board
LIT: Lupus Impact Tracker
LupusPRO: Lupus Patient Reported Outcome questionnaire
LupusQoL: Lupus Quality of Life questionnaire
PRO: patient-reported outcome
PROMIS^®^: Patient Reported Outcomes Measurement Information System
SD: standard deviation
SELENA-SLEDAI: Safety of Estrogens in Lupus Erythematosus National Assessment-Systemic Lupus Erythematosus Disease Activity Index
SF-36: Short Form-36
SIQ: SLE Impact questionnaire
SLAQ: Systemic Lupus Activity Questionnaire
SLE: systemic lupus erythematosus
SLEQOL: SLE-specific Quality of Life
SLICC/ACR: Systemic Lupus International Collaborating Clinics/American College of Rheumatology
SSD: SLE Symptom Severity Diary
